# Climate variables effect on fruiting pattern of Kinnow mandarin (*Citrus nobilis* Lour × *C. deliciosa* Tenora) grown at different agro-climatic regions

**DOI:** 10.1038/s41598-021-97653-1

**Published:** 2021-09-13

**Authors:** Rab Nawaz, Muhammad Azam Khan, Ishfaq Ahmad Hafiz, Muhammad Faisal Khan, Azeem Khalid

**Affiliations:** 1 Department of Horticulture, Pir Mehr Ali Shah- Arid Agriculture University, Rawalpindi, Pakistan; 2 Department of Environmental Sciences, Pir Mehr Ali Shah- Arid Agriculture University, Rawalpindi, Pakistan

**Keywords:** Ecology, Plant sciences

## Abstract

Kinnow orchards grown in different agro-ecological regions of Punjab, Pakistan, namely Sargodha, Toba Tek Singh (TTS) and Vehari districts, were selected to assess the effect of climate variables on fruit-bearing patterns. Experiment was laid out in RCBD while selecting identical features Kinnow plants and labeled twigs at analogous canopy positions in all three sites. Temperature was reported higher in TTS and Vehari areas, while relative humidity in Sargodha accounted for different levels of agrometeorological indices by computing more variations in warm districts. Climate variables influenced fruit-bearing habits and vegetative growth trend in all three flushes while recording heavy fruit-bearing plants during on-year and light fruit-bearing in off-year at Vehari. Similarly, three vegetative flushes were recorded unevenly in all three sites due to different fruit-bearing patterns induced by climate variables. Harvesting pattern of orchards began earlier in Sargodha, where maximum orchards were harvested before new flowering to add evenness to fruiting habits during on & off-years. In warm conditions, fruit ripening arrived in the peak of winter and mostly domestic market-driven harvesting resulted in late start of fruit picking with more erratic fruit-bearing habits. Both physiological and pathological fruit drops have been significantly affected by climate variables with a higher degree of physiological drop in warm regions and pathological effects in the humid conditions of Sargodha on heavy fruit-bearing plants. Fruit yield and grading quality were also affected in both seasons by showing more asymmetrical trend in yield and fruit grading in warm areas of TTS and Vehari due to an irregular fruiting pattern compared to Sargodha. From now on, the climate variables of the three sites directly influenced the fruiting patterns, vegetative flushes, fruit drops, yields and grades of Kinnow mandarin.

## Introduction

Climate variables of a particular area decide cropping system as well as agrometeorological/ thermal indices and energy use efficiencies^1,2^ by showing a fluctuating trend location-wise^3^ to count different levels in unalike crop in a specified region^4^. As climate variables of an agro-ecological zones depicts temperature regimes, relative humidity, sunshine duration, solar radiation intensity, precipitation and wind velocity^5,6^ henceforth, are used to compute thermal indices^7^ like growing degree days (GDDs), crop/ citrus heat unit (CHU), modified citrus heat unit (mCHU), hydrothermal units (HYTUs), photothermal index (PTI), photo thermal unit (PTU) and helio thermal unit (HTU)^4,8,9^. Thermal indices decide citrus fruit phenophases, peel coloration, physico-chemical quality, abiotic & biotic stress, maturity indices as well as grading volume^6,10–12^, while indirectly fruit-load and net-return^13^, fruit cosmetic outlook^14^, harvesting and marketing^15^ as well as consumers penchant^16^. More extremes in climate variables are seen in global warming era due to rising temperatures^17^ which have increased abiotic and biotic stress^18^ while escalating pests pressure^19^ in temperate^20^ and subtropical zones^21^ and showing more unevenness in changing weather conditions^22^ to negatively affecting perennial crops, including citrus crop as their slow acclimatization^23^. Therefore, climate variables have a direct effect on the growth stages of citrus^24^, external outlook^25^, quality and bearing habits^26^ by showing further fluctuations in unalike ecological zones^27^. Similarly, asymmetry in fruiting habits is shown in extreme climate variables^28^ by displaying more differences in its magnitude in different regions^29^ with more irregularity in warm areas^13^. Climate variables depict phototemperature (Tp), nyctotemperature (Tn), relative temperature and humidity disparities, photo & nycto humidity levels as well as vapor pressure deficit (VPD) by using temperature and relative humidity for these variables computation^30–32^ are further utilized in plant adoptive behavior, growth and development and survival against erratic weather^33^ to predict weather conditions^34^ suitability for certain crop in a particular area^35^ and finally reproductive span and behavior^36^.

Biennial fruiting habit is heavy and light fruit-load in the alternative season^28^ is an inherent tendency in citrus^37,38^, particularity in mandarin^39^. The relationship between the citrus plant source (leaves) and the sink (fruits & roots) determines the vegetative and floral growth trend^40^ which has become imperfect in heavy fruit-bearing plants^41^ to slow down the growth of root and aerial parts, particularly in mandarin, in order to induce biennial fruiting habit^42^. In addition, heavy fruit-load plants source (leaves) photoassimilates are more streamlined towards fruits^43^ which has depleted carbohydrates to the rest of the plant parts including aerial (shoots & leaves) and ground (roots) to induce low floral bud induction for the coming season^44^ is typically seen in citrus^45^, avocado^46^, olive^47^ and mango^48^. Fruit-load specifically disrupts amino acids, coenzymes and sugars^49^ and also the phyto-inhibiting effect on incoming flowers^50^ to stimulate uneven fruiting patterns^38^. Heavy fruiting during on-year leave cyclical carry-over effects that minimize carbohydrates^51^ and also string inhibitory effect of phytohormones on flowering during the off-year period^50^ by endorsing nutritional and hormonal aspects on citrus with annual fruiting pattern^52,53^. Biennial fruiting tendency causes fruit loss^54^, disrupts the supply-chain process^55^ and decreases orchard production and profit^56^, resulting in a marketing failure^57^.

King (*Citrus nobilis* Lour) used as a seed and Willow (*C. deliciosa* Tenora) as a pollen parent to evolve Kinnow, an F1 hybrid generation by H.B Forest, a citrus breeder at Citrus Research Institute, University of California, Riverside, the USA, was gifted to Queen Victoria and introduced in Sub-continent in 1942 during Colonial regime, had heightened citrus industry of Pakistan with sole dominancy in export^15,58^. Among citrus cultivars, it has a dominant share, mainly growing in the Punjab plain and exclusive citrus fruit, exported to the world^13^. The same citrus cultivar, including Kinnow mandarin, behaves differently under uneven agro-climatic conditions due to oscillation in abiotic and biotic stress^11^, changes in fruit growth, development and ripening^10^ as well as quality and harvesting patterns^13,15^. Present work was conducted in three different Kinnow growing zones to assess the effects of climate variables on fruit-bearing habits by selecting three sites in the districts Sargodha, TTS and Vehari in the province of the Punjab, Pakistan.

## Materials and methods

This study was carried out in the plains of Punjab, Pakistan, by selecting three experimental sites in different agro-climatic zones located in Sargodha, TTS and Vehari during the Kinnow orchard growing seasons 2017–2018 (on-year) and 2018–19 (off-year).

### Selection of orchards

Kinnow orchards were selected in block form with similar characteristics of plants like age, health, vigor, planting system/ geometry (square), density (250–260 plants/ha) and grafted on Rough lemon(*Citrus Jambhiri* Lush.) rootstock^10^ by tagging branches/ twigs to reflect uniform canopy positions of the individual plant^11^. Basic soil properties were analyzed in three sites with organic matters ranges (0.75–0.80%), available phosphorous (6.0–6.5 mg kg^−1^), available potassium (230–260 mg kg^−1^) and loamy structure soil^11^. In each orchard, uniform dose of fertilizers i.e., Nitrogen (1000 g), phosphorous and potash (500 g each) were applied.

#### Plant material identification and not deposit in herbarium

The plants of Kinnow mandarin were selected by researcher (R.N) as being used in research. Plants having age 12–15 years old were not deposited in any public herbarium as it is not a new species, with no need to deposits as from orchard not uprooted.

### Climatic/weather data

**Figure 1:**
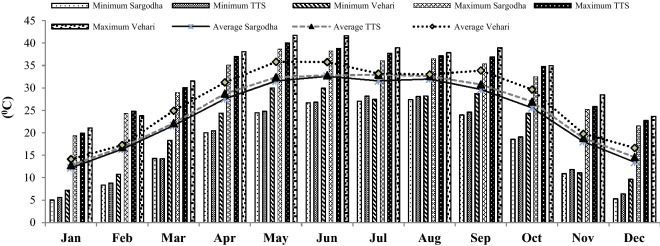
Temperature data of three districts.

**Figure 2:**
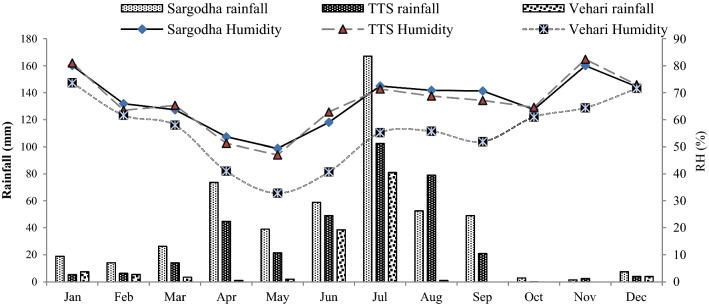
Weather data (rainfall and average relative humidity) of three districts.

Weather data were collected from the Pakistan Meteorological Department (PMD), Islamabad, of three experimental sites and the office of the Deputy Director Agriculture (Extension), Vehari, used in computation of climate variables. Temperature data are shown in Fig. 1 and rainfall as well as relative humidity in Fig. 2. Total annual precipitation (511, 349 and 144 mm), average annual temperature (23.65, 25.19 and 27.11 °C) and average annual relative humidity (66.8, 63.20 and 55.6 percent) were reported in the districts of Sargodha, TTS and Vehari during 2017 and 2018 respectively.

### Climate variables computation

Climate variables like relative temperature disparity (RTD), phototemperature (T_*p*_), nyctotemperature (T_*n*_), relative humidity disparity (RHD), photo relative humidity (RH_*p*_), nycto relative humidity (RH_*n*_), vapor pressure deficit (VPD), photo vapor pressure deficit (VPD_*p*_) and nycto vapor pressure deficit (VPD_*n*_) were calculated from weather data using computation of^30–32,59^. Relatively humidity recorded at midnight (0000 UTC) and midday (1200 UTC) was used while computing climate variables.

(i) RTD (%) = (T_max_ − T_min_)/T_max_ × 100
(ii) T_*p*_ (^o^C) = T_max_ − ¼ (T_max_ − T_min_)
(iii) T_*n*_ (^o^C) = T_min_ + ¼ (T_max_ − T_min_)
(iv) RHD (%) = (RH _0000 UTC_ − RH _1200UTC_)/RH _0000UTC_ × 100
(v) RH_*p*_ (%) = RH _1200UTC_ − ¼ (RH _1200UTC_ − RH _0000UTC_)
(vi) RH_*n*_ (%) = RH _0000UTC_ + ¼ (RH _1200UTC_ − RH _0000UTC_)
(vii) VPD = (es − e)/1000, where e = 6.11 × 10{7.11 × T_mean_ /(237.3 + T_mean_)} and es = e (100 − RH _mean_)
(viii) VPD_*p*_ = (es − e)/1000, where e = 6.11 × 10{7.11 × T_*p*_/(237.3 + T_*p*_)} and es = e (100 − RH _1200UTC_)
(ix) VPD_*n*_ = (es − e)/1000, where e = 6.11 × 10{7.11 × T_*n*_/(237.3 + T_*n*_)} and es = e (100 − RH _0000UTC_)

### Thermal indices computation

Thermal indices like growing degree days (GDDs), citrus heat unit (CHU), modified citrus heat unit (mCHU), hydrothermal units (HYTUs), photothermal index (PTI), helio thermal unit (HTU), photo thermal unit (PTU), phototemperature heat unit and nyctotemperature heat unit were computed from three experimental sites^5,8–10,60^. Modified citrus heat unit (mCHU) derived using nyctotemperature (T_*n*_) as minimum temperature and phototemperature (T_*p*_) as maximum temperature in calculation.

(i) GDDs = (T_max_ + T_min_)/2 − T_base,_ wherein citrus T_base_ is 13 °C.
(ii) CHU = (X + Y)/2 where X = 1.8(T_min_ − 13) and Y = 3.3(T_max_ − 13) − 0.083(T_max_ − 13)^2^
(iii) mCHU = (X + Y)/2 where X = 1.8(T_*n*_ − 13) and Y = 3.3(T_*p*_ − 13) − 0.083(T_*p*_ − 13)^2^
(iv) HYTUs = GDDs x mean RH (individual day)
(v) PTI = GDDs / No. of days (Fruit-set to maturity)
(vi) PTU = GDDs x day length in hours
(vii) HTU = GDDs x bright sunshine hours
(viii) *p* Heat unit = Phototemperature (T_*p*_) − T_base_
(ix) *n* Heat unit = Nyctotemperature (T_*n*_) − T_base_

### Thermal energy use efficiency

Thermal energy use efficiency was computed through Kinnow plant yield (kg ha^−1^) basis. Accumulated thermal indices used in computation of thermal energy use efficiency were calculated from fruit-set till arrival of maturity in fruit^2,5,9–11^.

(i) Heat use efficiency = Yield (kg ha^−1^)/accumulated GDDs
(ii) Heliothermal use efficiency = Yield (kg ha^−1^)/accumulated HTU
(iii) Photothermal use efficiency = Yield (kg ha^−1^)/accumulated PTU
(iv) Hydrothermal use efficiency = Yield (kg ha^−1^)/accumulated HYTUs
(v) *photo* Heat use efficiency = Yield (kg ha^−1^)/accumulated *p* Heat unit
(vi) *nycto* Heat use efficiency = Yield (kg ha^−1^)/accumulated *n* Heat unit

### Measurement of bearing habit

Randomly 10 lines with 10 plants in each line were tagged to count the bearing habit at each experimental site. Both seasons, fruiting habits were counted by recording light, medium and heavy bearing-plants and value in percentage was calculated from total labeled trees.

### Flush quantification

A quadrate of scale (0.5 × 0.5 × 0.5 m^3^) was used to quantify three vegetative flushes by counting number of twigs and leaves per twig inside the quadrate.

### Orchard harvesting trend

A survey was conducted in three districts to assess the harvesting trend of Kinnow orchards. Maturity indicators in commercial orchards are mostly based on peel yellow-orange coloration and reduce in acidity with sweet taste. While in present work, in addition to peel coloration, Refractometer is used to measure total soluble solids (TSS) with its value above 10^o^Brix as being used as internal maturity indicator which arrive mostly on external peel complete yellow coloration.

### Fruit drop trend

Physiological fruit drop at the end of June and pathological drop at the end of December, when the fruit matured, were counted from the tagged branches of the selected plants.

### Fruit yield and grades percentage

At harvest time, yield and fruit grading parameters were used to measure the fruit weight, number and fruit grading of the selected plants.

### Statistics design

The research was designed in randomized complete block design using factorial analysis on the Statistix 8.1 software. Analysis of variance of the individual parameter was performed using LSD while keeping P value at P ≤ 0.05. In tables data are shown in means ± standard deviation (M ± SD).

### Code of ethics

No human or animals involved in this experiment. Kinnow plants in the present study comply with international, national and/or institutional guidelines. No extinct species or new used and not deposit in herbarium.

## Results and discussion

A significant difference in fruit bearing habits was seen during the on- & off-year period, which is explained as below.

### Climate variables of experimental sites

Relative temperature disparity (RTD) is wider in winter season and narrows down in summer with a higher trend in Sargodha and lower in Vehari. Phototemperature recorded from daylight maximum temperature and nyctotemperature to depict night duration lower temperature and both showing an increasing level in warm conditions of Vehari during summer and lower at Sargodha in winter months. However, higher phototemperature was reported in Vehari (40.1 °C) in June 2018 and lower nyctotemperature was observed in Sargodha (8.0 °C) in January 2018. Relative humidity disparity (RHD) is the difference between the relative humidity reported at midnight (0000 UTC) and midday (1200 UTC) with widening differences between April and May in both years in Sargodha, followed by TTS and narrowed down in August–September in all three districts. Photo relative humidity (RHp) recorded at midday showed higher trends in November–December and lower during April–May in both seasons and all three districts. Nycto relative humidity (RHn) was measured from midnight, showing a rising trend in December-January and a declining trend in May–June for both seasons and three locations. However, RH*p* was recorded higher in TTS (77%) in November 2018 and lower at Vehari (22.26%) in May 2018. Alike RH*p*, nycto RH was also recorded higher at TTS (87.80%) in November 2018 and lower in May 2018 at TTS (30.71%). Vapor pressure deficit (VPD) was calculated using temperature and relative humidity regimes, showing an increasing trend in warm months with low RH levels and higher VPD was computed in May 2018 in Vehari (4.50 kPa) and lower in January 2017 in Sargodha (0.40 kPa). Photo VPD computed by using phototemperature and midday time recorded RH, showing higher levels in warm conditions of Vehari and TTS during dryer months of April–May and lower in winter month. Like VPDp, nycto VPD was calculated using nyctotemperature and midnight RH with the same rising trend in warm regions in April–May and decreased during winter months. VPD*p* was recorded higher in May 2018 at Vehari (4.78 kPa) while VPD*n* was recorded as lower during January 2017 at TTS (0.17 kPa). Data are shown in Table 1.

**Table 1:** Climate variables of experimental sites.

Variables	Climate variables of three experimental sites													
	Sites	Year	Jan	Feb	Mar	Apr	May	Jun	Jul	Aug	Sep	Oct	Nov	Dec
RTD (%)	SGD	2017	63.68	63.60	51.55	42.67	35.80	31.02	24.15	26.07	32.78	43.19	54.85	74.61
		2018	81.18	67.70	50.33	42.21	37.17	28.53	25.92	23.99	31.78	43.13	57.31	74.92
	TTS	2017	64.02	64.40	55.50	44.96	36.78	31.59	25.42	24.73	34.98	46.02	49.73	71.12
		2018	76.17	64.88	50.75	44.32	39.29	29.44	25.48	23.94	31.70	44.68	55.98	71.84
	VEH	2017	64.99	53.01	42.05	36.75	30.94	30.56	32.39	25.54	27.39	31.16	61.00	59.40
		2018	66.40	57.64	42.05	34.62	25.48	25.54	26.66	25.54	25.20	31.16	61.00	59.40
T *p* (^o^C)	SGD	2017	15.0	20.2	24.1	31.6	35.4	35.3	34.3	34.1	32.1	30.0	20.5	18.0
		2018	16.5	20.4	26.5	30.8	34.8	35.5	33.4	34.5	32.9	28.1	22.7	17.0
	TTS	2017	15.2	21.0	25.1	33.0	36.8	35.3	35.6	34.8	34.2	31.6	20.7	18.9
		2018	17.4	20.6	27.1	32.8	35.6	36.2	35.1	35.0	33.5	30.1	24.0	18.4
	VEH	2017	16.1	20.5	28.2	35.0	37.9	37.3	35.0	35.5	35.3	32.3	24.1	20.2
		2018	19.1	20.6	28.2	34.3	39.7	40.1	37.2	35.5	37.6	32.3	24.1	20.2
T *n* (^o^C)	SGD	2017	9.2	12.6	17.1	24.0	28.4	29.3	29.9	29.3	26.4	22.8	13.8	9.6
		2018	8.0	12.1	18.8	23.5	27.6	29.9	28.7	30.1	27.2	21.4	15.1	9.2
	TTS	2017	9.2	12.9	17.2	24.6	29.3	29.2	30.8	30.2	27.6	23.5	14.5	10.5
		2018	9.2	12.7	19.2	24.6	27.8	30.4	30.3	30.5	27.8	22.5	16.2	10.4
	VEH	2017	9.8	14.3	21.6	27.9	31.5	31.1	28.8	30.6	30.1	27.0	15.4	13.1
		2018	11.5	13.7	21.6	27.8	34.3	34.6	31.9	30.6	32.5	27.0	15.4	13.1
RHD (%)	SGD	2017	25.00	41.74	39.43	46.19	41.41	33.76	25.94	31.56	34.52	41.62	28.03	31.52
		2018	37.87	45.76	44.37	38.03	43.67	33.86	17.74	22.39	38.43	39.27	31.70	27.63
	TTS	2017	28.25	49.24	45.71	50.48	44.68	35.07	27.17	34.65	40.22	45.65	23.92	32.33
		2018	39.94	47.60	50.25	44.79	48.60	31.21	28.11	27.09	36.22	41.60	34.21	28.99
	VEH	2017	32.42	51.47	33.77	48.79	18.50	23.09	34.40	33.40	36.34	32.92	28.34	26.33
		2018	30.41	34.07	35.61	48.22	48.61	44.08	35.25	33.40	31.79	58.09	54.12	47.68
RH *p* (%)	SGD	2017	73.75	57.32	55.84	45.34	42.79	52.71	66.52	63.66	62.90	56.10	73.78	65.55
		2018	64.99	54.05	52.86	49.17	40.61	52.44	71.36	68.57	59.71	55.80	65.43	72.42
	TTS	2017	74.11	52.83	55.60	42.48	40.02	56.19	65.10	61.29	58.70	55.06	77.00	65.65
		2018	64.03	55.75	51.52	39.69	38.05	54.48	66.16	64.32	60.45	58.01	64.18	72.59
	VEH	2017	66.52	52.29	52.55	34.28	40.49	46.11	48.91	50.09	46.96	54.97	59.18	66.28
		2018	67.11	53.76	51.65	34.63	22.26	27.51	49.71	50.09	46.04	44.12	48.20	54.07
RH *n* (%)	SGD	2017	85.06	74.54	71.52	61.36	55.92	64.66	77.12	76.66	77.37	73.02	86.26	78.90
		2018	82.14	73.45	70.52	62.63	55.19	64.33	78.73	78.30	75.93	71.33	78.97	85.06
	TTS	2017	87.44	73.42	75.05	60.05	53.94	69.51	76.60	75.68	75.57	74.14	87.80	79.58
		2018	82.10	76.32	72.19	53.41	52.98	65.72	77.84	75.48	75.62	75.64	78.93	85.96
	VEH	2017	80.77	74.07	64.61	47.52	44.92	52.93	60.54	61.36	58.81	67.03	69.66	76.91
		2018	80.31	66.13	64.31	47.68	30.71	36.26	61.65	61.36	55.66	66.94	70.20	74.35
VPD (kPa)	SGD	2017	0.40	0.95	1.25	2.10	2.57	2.12	1.42	1.50	1.38	1.50	0.54	0.65
		2018	0.56	0.99	1.43	1.93	2.59	2.17	1.21	1.33	1.52	1.46	0.86	0.47
	TTS	2017	0.38	1.05	1.21	2.28	2.77	1.92	1.51	1.61	1.60	1.56	0.50	0.68
		2018	0.63	0.93	1.46	2.47	2.75	2.12	1.43	1.53	1.54	1.40	0.93	0.49
	VEH	2017	0.57	1.08	1.67	2.97	3.13	2.73	2.29	2.30	2.43	1.83	1.15	0.80
		2018	0.84	1.34	1.97	3.25	4.50	4.24	2.73	2.60	2.96	2.64	1.70	1.28
VPD *p* (kPa)	SGD	2017	0.65	1.45	1.76	2.76	3.18	2.63	1.80	1.95	1.88	2.10	0.89	1.06
		2018	1.00	1.56	2.04	2.51	3.27	2.68	1.49	1.69	2.08	2.00	1.28	0.78
	TTS	2017	0.66	1.65	1.82	3.02	3.45	2.45	1.93	2.11	2.21	2.25	0.79	1.10
		2018	1.09	1.51	2.15	3.14	3.47	2.62	1.85	1.94	2.08	2.01	1.39	0.83
	VEH	2017	0.89	1.63	2.15	3.63	3.51	3.14	2.81	2.76	2.93	2.28	1.59	1.14
		2018	1.03	1.58	2.20	3.56	4.78	4.50	2.91	2.76	3.14	2.85	2.04	1.53
VPD *n* (kPa)	SGD	2017	0.21	0.54	0.82	1.52	2.01	1.67	1.07	1.08	0.94	0.99	0.28	0.34
		2018	0.24	0.55	0.92	1.43	1.98	1.70	0.96	1.01	1.03	1.00	0.51	0.23
	TTS	2017	0.17	0.58	0.72	1.63	2.16	1.45	1.13	1.16	1.06	0.98	0.26	0.36
		2018	0.29	0.49	0.89	1.87	2.11	1.67	1.05	1.16	1.07	0.89	0.55	0.24
	VEH	2017	0.31	0.63	1.25	2.37	2.77	2.34	1.82	1.87	1.97	1.42	0.78	0.52
		**2018**	0.38	0.80	1.26	2.36	3.75	3.48	1.94	1.87	2.27	1.41	0.77	0.56

RTD (relative temperature disparity), T *p* (*thermo* temperature), T *n* (*nytco* temperature), RHD (relative humidity disparity), RH *p* (*photo* relative humidity), RH *n* (*nytc*o relative humidity), VPD (vapor pressure deficit), VPD *p* (*photo* vapor pressure deficit), VPD *n* (*nytco* vapor pressure deficit).

Different altitudes, longitudes and latitudes have variation in climatic conditions and all three experimental sites are located in three different agro-ecological and agro-climatic zones viz; Sargodha (32.0837°N, 72.6719°E) altitude 189 m, Toba Tek Singh (30.9727°N, 72.4850°E) altitude 161 m and Vehari (30.0452°N, 72.3489°E) altitude 140 m^10^. Sargodha district is to the north with a higher elevation, while Vehari is to the south with a low elevation. The climatic conditions are therefore different on a monthly as well as an annual basis in all three sites by having a direct effect on plant physiology^7^ and indirectly on fruit bearing habits and maturity arrival on Kinnow fruit^3,12^. Climate variables decide plant adaptation, developmental phase and thrive against vagaries of weather^33^, and also depict short and long weather conditions of a particular region^34^. Temperature has a cumulative effect on growth, yield and productivity span of plants^35,36^ as well as used in computation of climatic indices which are further utilized in management process^61,62^. Phototemperature is climate variable to depict a daytime temperature which also termed as active or positive temperature just above base temperature of particular crop in most days of the year^62,63^ and has been widely used in the measurement of different heat units^64,65^. Nyctotemperature derived from minimum temperature is referred as inactive temperature, which usually fall below the threshold temperature of sub-tropical crops like citrus in all winter, spring and autumn months except for a few summer months in warm regions^9,66^ and also decide on the accretion in heat units used in plant phenological studies as well as future strategies for controlling insect-pests and diseases^67^. Temperature directly affects plant tissues and organs with death in extreme conditions^68^ while its intermediate level affects physiological processes, including reproductive organs^69^ that cause imperfections in flower and fruit development^70^. Temperature-based climate variables influence fruit growth, quality and seasonal yield^71^ while temperature fluctuations directly affect citrus physiology^72^ and indirectly reduce yield^73^.

Temperature and relative humidity (RH) are used to compute vapor pressure deficit (VPD), is a difference in air saturation capacity (es) to actual water content/vapor (e) in air^74,75^, which is used to identify eco-physiology and hydraulic traits of plant growth^76^, since global warming has shown a fluctuating year-round pattern and a rising level^77^. VPD regulates stomatal conductance^78^ which triggers its cavity to open at lower and closer at higher levels^79^. Leaf to air VPD is widening in high temperature and low RH regime and vice versa, to affect photosynthesis and transpiration^74,80,81^, is also an influential tool to judge ecological behavior of a plant under varying environmental conditions^76^ while its fluctuation or elevation directly affects plant growth^82^. High VPD was recorded in the warm conditions of Vehari and TTS, especially in the starting months of summer season, to cause stress to Kinnow plant in both seasons, resulting in more fruit drops, which further developed unevenness in both fruiting seasons. In elevated VPD regime, more water loss due to evapotranspiration, resulting in plant-water imbalance^83^ causes plant physiological process malfunction^84^ with low carbon assimilation and high transpiration rates resulting in carbon starvation and hydraulic break-down^85^. VDP not only indicates temperature and RH regime of a particular area, but also dictates leaf stomatal conductance, transpiration rate, carbon assimilation, uptake of nutrients, plant-water hydraulic control and stress conditions^86,87^. It also controls gases exchange and defines stomatal limitation by recording a higher range in summer and a lower in winter^80,88–90^. However both RH and temperature different regimes decide the extent of diseases, pests and fruit quality of citrus^15^ by recording more pest damage in dry conditions and diseases infestation in warm-humid areas to affect fruit quality^11^ which has justified the present work of showing more unevenness in fruiting patterns in warm conditions due to more fluctuation in climate variables. Climate variables also influence on citrus external quality features based on color development^6^ as well as internal changes in the levels of juice contents, acidity, sugars and polyphenols^12^.

### Thermal indices and energy use efficiency

Growing degree days (GDDs) were counted as maximum in the warm district of Vehari (5076.5 °C day) and minimum in the district of Sargodha (3929.1 °C day) in 2018 season from fruit-set till arrival of maturity. In same season 2018, citrus heat unit (CHU) and modified citrus heat unit (mCHU) were computed higher in Vehari (7316.4 and 8170.3 °C day) and lower in Sargodha (6169 and 6862.1 °C day), respectively. Similarly, a higher photothermal index (PTI) was found at Vehari (17.09 °C) and lower one at Sargodha (13.79 °C) during 2018 season. However, hydrothermal units (HYTUs) accounted more for TTS (266,322 °C day %) in 2017 and less for Vehari region (233,308 °C day %) in 2018. Both photo and helio thermal units were accumulated more at Vehari (14,792,109 and 11,432,481 °C day hours) and less in Sargodha region (10,125,201 and 7,896,448 °C day hours), respectively during 2018. Phototemperature heat unit (pHU) and nyctotemperature heat unit (nHU) were computed by subtracting base temperature on daily basis and recorded higher in Vehari (5965.75 and 4208.75 °C day) and lower in Sargodha (4818.85 and 3113.23 °C day), respectively in 2018 season. Kinnow fruit-setting till arrival of maturity has taken maximum (297 days) at Sargodha in 2018 and minimum (282 days) in Vehari during 2017.

Thermal use efficiency or thermal energy use efficiency showed a higher trend in heavy-fruiting plants during on-year and lower in light-fruiting in off-year due to significant differences in Kinnow fruit yield. However, different heat use efficiencies were recorded as higher in TTS during on-year on heavy fruiting plants and lower at Vehari during off-year in light fruiting plants. Heat use efficiency, photo heat use efficiency, nycto heat use efficiency, all were recorded higher in heavy-fruiting plants during on-year at TTS (11.098, 9.013 and 14.168 kg °C day^−1^) and lower on light-fruiting plants during off-year at Vehari (1.862, 1.584 and 2.245 kg °C day^−1^), respectively. Helio and photothermal use efficiencies were recorded higher on heavy-fruiting plants during on-year at TTS (0.006 and 0.004 kg °C day^−1^ h^−1^), respectively and lower on light-fruiting plants during off-year in all three districts and both seasons (0.001 °C day^−1^ h^−1^ each). Similarly, hydrothermal use efficiency was computed higher on heavy-fruiting plants during on-year at TTS (0.179 kg °C day^−1^ h^−1^) and lowers with minute’s difference on light-fruiting plants in all districts and both on-& off-years. The data is shown in Tables 2 and 3.

**Table 2:** Thermal indices of experimental sites.

	Thermal indices of three experimental sites					
	Sargodha		T.T Singh		Vehari	
	2017	2018	2017	2018	2017	2018
GDDs (°C day)	4014.9	3929.1	4289.9	4255.7	4792.0	5076.5
CHU (°C day)	6249.0	6169.0	6391.5	6437.4	7117.1	7316.4
mCHU (°C day)	6996.0	6862.1	7269.0	7270.1	7992.7	8170.3
HYTUs (°C day %)	250,786	248,275	266,322	262,059	249,051	233,308
PTI (°C)	13.89	13.79	14.64	14.73	16.03	17.09
PTU (°C day hours)	10,641,199	10,125,201	11,878,037	11,731,858	14,020,769	14,792,109
HTU (°C day hours)	8,521,921	7,896,448	8,344,101	8,287,976	10,216,209	11,432,481
*p* Heat unit (°C day)	4940.275	4818.85	5282.25	5223.875	5734.5	5965.75
*n* Heat unit (°C day)	3186.925	3113.225	3360.35	3355.7	3866	4208.75
Period (Fruit-set to maturity)	292 days (18/03 to 31/12)	297 days (23/03 to 31/12)	288 days (14/03 to 31/12)	292 days (18/03 to 31/12)	282 days (08/03 to 31/12)	284 days (10/03 to31/12)

GDDs (growing degree days), CHU (citrus heat unit), mCHU (modified citrus heat unit), HYTUs (hydrothermal units), PTI (photothermal index), PTU (photo thermal unit), HTU (helio thermal unit), p (photo period), n (nytco period).

**Table 3:** Thermal use efficiency of Kinnow plant in biennial bearing pattern.

	Fruiting habit plants	Thermal use efficiency					
		Sargodha		T.T Singh		Vehari	
		On-year (2017)	Off-year (2018)	On-year (2017)	Off-year (2018)	On-year (2017)	Off-year (2018)
Heat use efficiency (kg °C day^−1^)	Heavy fruiting	8.508	7.723	11.098	9.677	6.542	4.893
	Medium fruiting	5.221	4.878	6.976	6.150	3.934	3.555
	Light fruiting	2.471	2.347	2.654	2.333	1.928	1.862
Heliothermal use efficiency (kg °C day^−1^ h^−1^)	Heavy fruiting	0.004	0.004	0.006	0.005	0.003	0.002
	Medium fruiting	0.002	0.002	0.004	0.003	0.002	0.002
	Light fruiting	0.001	0.001	0.001	0.001	0.001	0.001
Photothermal use efficiency (kg °C day^−1^ h^−1^)	Heavy fruiting	0.003	0.003	0.004	0.004	0.002	0.002
	Medium fruiting	0.002	0.002	0.003	0.002	0.001	0.001
	Light fruiting	0.001	0.001	0.001	0.001	0.001	0.001
Hydrothermal use efficiency (kg °C day^−1^%)	Heavy fruiting	0.136	0.122	0.179	0.157	0.126	0.106
	Medium fruiting	0.084	0.077	0.112	0.100	0.076	0.077
	Light fruiting	0.040	0.037	0.043	0.038	0.037	0.041
*Photo* Heat use efficiency (kg °C day^−1^)	Heavy fruiting	6.915	6.297	9.013	7.884	5.467	4.164
	Medium fruiting	4.243	3.978	5.665	5.010	3.288	3.025
	Light fruiting	2.008	1.914	2.155	1.900	1.611	1.584
*Nycto* Heat use efficiency (kg °C day^−1^)	Heavy fruiting	10.719	9.747	14.168	12.272	8.109	5.902
	Medium fruiting	6.577	6.157	8.905	7.799	4.876	4.287
	Light fruiting	3.113	2.962	3.388	2.958	2.390	2.245

Temperature counted directly GDDs, different heat units and indirectly PTI, HYTUs, PTU and HTU of a specific region on the basis of the threshold temperature of certain crops^11,60,91^. Citrus growth ceases below 13 °C^92^ by keeping this temperature as threshold when computing growing degree days (GDDs) and other heat units of subtracting base temperature from mean daily temperature^10,93^. Vehari region has higher mean daily temperature, followed by TTS and lower was recorded in Sargodha^15^, accordingly GDDs, CHU, mCHU, p Heat unit and n Heat unit were counted in all three districts with leading counts in Vehari and lesser in Sargodha^6,12^. More GDDs were counted of citrus fruit in the warm areas of Australia^92^ to justify this work of recording additional GDDs at warm district Vehari along with other temperature based heat units such as CHU, mCHU, pHu, nHu. Similar results were reported by^94,95^ of counting more GDDs in warm regions due to the observation of higher average daily temperatures. Citrus fruit different growth phases counted different levels of GDDs^10^ but temperature directly affects GDDs and other heat units and also determines the occurrence of phenophases of fruit^9^. More GDDs, CHU, mCHU, pHu and nHu were counted in the warm region of Vehari, thus influencing fruit growth and development from fruit-setting till arrival of maturity and subsequently the fruiting habit of the Kinnow mandarin.

Hydrothermal units (HYTUs) were computed directly from average relative humidity (RHa) by multiplying with GDDs^9,11^ and recorded more in warm-humid regions like TTS^6,12^. In this work, the GDDs were more computed at Vehari around the year, but with low level of RHa and vice versa in the case of Sargodha region, henceforth the counts of HYTUs in these two districts were lower than those of TTS. However, a fluctuating trend in HYTUs counts has been seen in three districts from fruit-setting to maturity due to climate variables supported by the work of^9^ on Kinnow mandarin in India to record different HYTUs counts during fruit development. There was an increasing trend in HYTUs count during the summer season due to high daily means temperature and RHa during the rainy season compared to the rest of the months^12^. A fluctuating degree of HYTUs has been seen in all three districts due to the fact that they are located in different agro-ecological and agro-climatic areas, affecting the growth and development of Kinnow fruit and ultimately affecting fruiting habits as well as harvesting patterns. Similar results have been found^5,9^ in India.

Photothermal index (PTI) is directly computed from mean daily temperature divided by time span in particular regions^5,96^ and varies in different growth phases of citrus fruit^9^ by showing a fluctuating trend across the year and locations^6^. In present work, PTI was more counted in warm regions and also in summer months due to higher mean daily temperature as seen in the Vehari district. PTI indicates daily photothermal index of a particular area^9^ and can show a fluctuating trend in fruit different phenophases by recording more in cell division and cell enlargement stages^10^ due to elevated temperature when Kinnow fruit these stages are continue, especially in summer months. PTI directly decides the span of citrus fruit different growth phases and indirectly fruit-setting and harvesting trend^10^ and eventually bearing habit.

Photo and Helio thermal units reflect day-length, bright sun-light period and temperature levels of specific area and are directly computed by multiplying GDDs with day length and bright sunshine hours^5,9^. PTU and HTU were recorded more in warm regions and also in summer period due to elevated temperature and additional day-length than the cool season in the winter months^6^. Similar findings were seen in present work with additional PTU and HTU counts in Vehari district in both seasons and lesser in Sargodha from fruit-set till maturity. Same results of fluctuating PTU and HTU levels in different plum genotypes were reported by^5^ in different climatic conditions of semi-arid regions in India to endorse this work. Climate variables in the three districts have distinct differences in mean daily temperature and length of the day; as a result, major changes have been seen in their monthly and annual counts. Climate variables decides meteorological indicators or thermal indices of a certain crop grown in a particular regions^2,4^ to directly influence citrus fruit-setting, growth and development phases^10^, quality and yield features^11^, color-break and color development^6^ as well as biochemical properties and maturity index^12^. Similarly, climate variables also decides fruit-setting time^9^, maturity arrival^3^ and harvesting and marketing of Kinnow fruit^15^ which significantly contributed to the fruiting pattern to justify this work.

Thermal/ energy use efficiencies are computed directly on yield basis by dividing thermal indices to depict higher levels in high yielding crop and low thermal indices regions^2,96^ by indicating positive yield correlation and inversely with different heat units accretion based on temperature, RHa, bright sun-light and day-length^4,9^. Temperature and RH based thermal use efficiencies were recorded higher in TTS during on-year on heavy-fruiting plants due to added yield but more thermal indices than Sargodha where yields were low. Although, thermal indices were counted more at Vehari except HYTUs but yield on all three fruiting habit plants was less during both on-& off-years than TTS and Sargodha. Similar findings regarding low yield and higher agrometeorological indices to computed lesser energy use efficiency was reported by^9^ and^4^ to support present work of calculating lower thermal use efficiency on low yielding plants and districts. More yields on heavy-fruiting plants during on-year were recorded in all three sites, henceforth, energy use efficiencies were computed to be higher than light-fruiting plants in off-year period to induce irregular bearings and further fluctuations in fruiting habits were caused by extreme climate variables which adversely affected plant physiology and tree potency in uneven fruit bearings.

### Fruiting habit/fruit-bearing trend of plants at experimental sites

Heavy fruiting plants were recorded as maximum in on-year (35%) and minimum in off-year (9%) at Vehari. However, medium fruiting plants were counted more in Sargodha (65%) and less in Vehari (45%) during the on-year. Similarly, light fruiting plants were found more on an off-year basis (41%) and less on an on-year basis (20%) in Vehari. Data are shown in Table 4, indicating significant differences in both the three experimental sites and the on- & off-years fruiting patterns.

**Table 4:** Fruiting habit of plant of experimental sites during on- & off-years.

	Fruit bearing habit of Kinnow plants at Experimental sites					
	Sargodha		T.T Singh		Vehari	
	On-year	Off-year	On-year	Off-year	On-year	Off-year
Heavy fruiting plants %	15 ± 3.35j	10 ± 2.35k	30 ± 4.25g	10 ± 1.56k	35 ± 3.15f	9 ± 3.25k
Medium fruiting plants %	65 ± 4.01a	50 ± 4.5c	49 ± 4.25c	55 ± 4.25b	45 ± 4.25d	50 ± 4.36bc
Light fruiting plants %	25 ± 2.44h	40 ± 3.78e	21 ± 3.5i	35 ± 3.25f	20 ± 2.25i	41 ± 2.14de

Results are shown in means (± SD) and sharing different letters are significantly differed to each other according to LSD test (P ≤ 0.05).

Fruiting patterns of citrus plants are directly influenced by environmental conditions^13^, cultivars with a bearing habit either on a single plant or on any branch or in a whole cluster / block form^28^ thus exhibiting a superfluous predisposition in the mandarin group^37,39^. Heavy fruiting plant depletes carbohydrates and leaves low photoassimilates for next season flowering or fruiting^44^ which imposes competition for carbohydrates and eventually resulted in alternate bearing habits in citrus^45^, mango^48^ and avocado^46^. Harvesting time decides next season crop^97^ while timely harvesting of heavy fruited plants induces evenness in fruit-bearing habit^29^. In this work, Kinnow plants were harvested late in warm conditions due to inland market consumption and late arrival of maturity indicators for the choice of the native consumers, which resulted in more disproportion of fruiting patterns in the Vehari and TTS districts. On the other hand, early harvesting and spot-picking began in the Sargodha area, which had reduced on-tree fruit load by controlling symmetry patterns in fruit-setting and fruit-bearing habits for both on-and off-year fruiting seasons. In addition, extreme climatic variables also prompt asymmetry in the fruiting pattern^28,29^ and its magnitude was more recorded in warm regions^15^ to substantiate the present work implying unevenness in Kinnow plant fruiting in Vehari and TTS.

### Vegetative flush trend in different fruiting habit plants

Kinnow plants have three vegetative flushes, like other citrus cultivars, which showed a highly significant difference amongst spring, summer and autumn flushes and a slight difference in orchards grown in three different agro-climatic zones, as shown in Table 5. In the spring, vegetative flush was quantified as maximum on heavy-fruiting plants at Sargodha (60%) during on-year and minimum on light-fruiting plants at TTS (48%) in off-year. Maximum summer flush counts were recorded at TTS (35%) on light-fruiting plant during off-year and minimum at Sargodha (28%) on heavy-fruiting plant in on-year. However, the higher autumn flush was counted at TTS (18%) on light-fruiting plants in off-year and the lower on heavy-fruiting plants in Sargodha (12%) during on-year.

**Table 5:** Three vegetative flushes trend in different fruiting habits plants during on- & off-years.

Three vegetative flushes	Fruiting habit plants	Vegetative flush trend in different fruiting habit plants					
		Sargodha		T.T Singh		Vehari	
		On-year	Off-year	On-year	Off-year	On-year	Off-year
Spring flush (%)	Heavy fruiting	60 ± 2.13a	55 ± 2.15b	55 ± 1.98b	50 ± 2.22c	57 ± 2.41ab	53 ± 2.1b
	Medium fruiting	58 ± 2.35a	56 ± 1.93b	53 ± 3.21b	52 ± 2.85bc	56 ± 2.54b	55 ± 1.98b
	Light fruiting	55 ± 2.5b	57 ± 2.28ab	54 ± 2.41b	47 ± 3.05c	54 ± 2.74b	52 ± 2.32bc
Summer flush (%)	Heavy fruiting	28 ± 2.45e	29 ± 2.08e	30 ± 1.74e	34 ± 1.87d	30 ± 2.25e	32 ± 1.9de
	Medium fruiting	29 ± 2.21e	30 ± 2.23e	34 ± 3.15d	33 ± 2.73d	30 ± 2.16e	32 ± 1.24de
	Light fruiting	32 ± 3.22de	29 ± 2.62d	32 ± 3.15de	35 ± 3.03d	31 ± 3.16e	34 ± 3.21d
Autumn flush (%)	Heavy fruiting	12 ± 3.05g	16 ± 2.14f	15 ± 2.01f	16 ± 2.14f	13 ± 2.41g	15 ± 1.87f
	Medium fruiting	13 ± 1.98g	14 ± 2.23fg	13 ± 1.25g	15 ± 2.13f	14 ± 1.85fg	13 ± 1.24g
	Light fruiting	13 ± 2.21g	14 ± 2.33fg	14 ± 2.65fg	18 ± 2.43f	15 ± 2.2f	14 ± 3.15fg

Results are shown in means (± SD) and sharing different letters are significantly differed to each other according to LSD test (P ≤ 0.05).

Kinnow plants like rest of citrus cultivars have more than half percentage of spring, one-third summer and one-sixth autumn flushes^98^. In this work, spring vegetative flush was recorded higher in on- & off-years in all three fruiting habits plants and less was recorded in the autumn season. Similar findings of less summer flush than spring were attributed to harsh environmental conditions^99^. In all three districts, summer vegetative flush was also low than spring due to harsh external conditions, as well as on-tree fruits competed for carbohydrates during the summer months, when fruit cell division and cell enlargement stages were ongoing, with fruit expanding to maximum size^10^. Prior completion of cell division with the earlier commencement of the cell enlargement stages was observed in warm conditions^100^ to justify this work of relatively more quantification of summer flush in warm districts Vehari and TTS. In addition, GDDs, PTI, PTU and HTU were accrued as higher in Vehari and TTS during cell division stage, thus this stage earlier wrap up its process than Sargodha and then begin prior cell enlargement^10^. Reported^43^ that large chunks of photoassimilates were consumed during fruit growth and development in heavy-fruiting plants, while minutes share was streamlined towards vegetative growth to endorse this study of quantifying less spring and summer vegetative flushes on heavy-fruiting plants. The source-sink relationship for carbohydrates also defines the reproductive and vegetative growth habits of citrus plants^40,41^, while the fruit-load also restricts vegetative and root growth in mandarin to impute alternative bearings^42^.

In the off-year era, fewer carbohydrates consumed by fruit for growth and development, hence, more vegetative flush was recorded in light-fruiting plant during the off-season period and vice versa in heavy-fruiting plants during the on-year. In the off-season, fewer carbohydrates were consumed; thus, the next season (on-year) spring vegetative flush was recorded as more in all three experimental sites in all fruiting habit plants. Similar findings of developing fruits compete for carbohydrates in citrus^101^, which also justify this study. In autumn season, meteorological indices like GDDs, HYTUs, PTI, PTU and HTU were less available to Kinnow plant in all three sites due to low means daily temperature with day-length squeezing by slowing down the net photosynthesis rate and retarding vegetative growth, wherein less photoassimilates synthesized with low carbohydrates accumulation in plant parts, although on-tree hanging fruits were in maturing and ripening phase to compete less for carbohydrates. These findings are in line with the work of^52,102,103^. Vegetative flush quantification of Kinnow mandarin in this work endorsed the nutritional aspect of floral buds initiation regulated by fruit-load and availability of carbohydrates^45,51–53,104^ rather than phytohormones inhibitory effect on citrus flowering during on-year^50,52,102,105^.

### Harvesting trend of Kinnow orchards

Harvesting trend data for Kinnow orchards are shown in Table 6, which shows major variations in harvesting times as well as trends among three districts. Harvesting of Kinnow orchards was recorded as maximum during off-year period (46%) in Sargodha and minimum during on-year (20%) in Vehari at the end of December. Harvesting trend increased during off-year season at Sargodha (56 and 70%) and recorded low during on-year in Vehari (35 and 48%) in the midst of January and the end of January, respectively. Same increasing trend of orchard harvesting in on-year season was seen in Sargodha (85 and 92%) in mid and end of February, respectively. However, rapid harvesting began in February in both the TTS and Vehari districts, reaching over 80% during off-year period and above 70% in on-year season. In the mid-March, maximum orchards were harvested in Sargodha during the off-year (98%) compared to other districts in same period, slightly above 90%.

**Table 6:** Harvesting trend of Kinnow orchards at experimental sites during on- & off-years.

	Harvesting trend of Kinnow orchards at three experimental sites					
	Sargodha		T.T Singh		Vehari	
	On-year	Off-year	On-year	Off-year	On-year	Off-year
End of December	40 ± 4.25i	45 ± 3.24hi	25 ± 4.35k	28 ± 2.45k	20 ± 4.46l	26 ± 3.88k
Midst of January	47 ± 4.25h	56 ± 5.11g	38 ± 4.35ij	42 ± 2.25i	35 ± 5.11j	40 ± 5.21i
End of January	58 ± 3.25g	70 ± 3.44e	50 ± 4.15gh	55 ± 2.75g	48 ± 5.32 h	54 ± 5.18g
Midst of February	70 ± 3.25e	85 ± 5.34c	63 ± 5.15f.	70 ± 3.45e	60 ± 6.06 fg	66 ± 5.27ef
End of February	82 ± 2.95c	92 ± 4.21b	78 ± 4.29d	87 ± 4.22c	76 ± 4.35d	84 ± 4.52c
Midst of March	96 ± 1.84a	98 ± 3.02a	93 ± 3.24ab	96 ± 2.51a	90 ± 5.41b	96 ± 3.41a

Results are shown in means (± SD) and sharing different letters are significantly differed to each other according to LSD test (P ≤ 0.05).

In certain fruit crops, including citrus, a cyclical carry-over effects of previous year's fruit present on trees are dominantly competed for carbohydrate reserves^45^ and/ or trigger phyto-inhibiting effects on next season's floral bud-break/ initiation^50^ can stimulate irregular fruiting patterns, commonly called as biennial bearing^38^ while on-tree fruits influence on plant metabolism, like changes in coenzymes, sugars and amino acids, which are being accelerated when fruits are harvested late^49^. Similar findings of late harvesting in avocado induce biennial bearing with reduced fruit yields^106^. In this research endeavor, more alternative fruiting was recorded in TTS and Vehari due to delays in harvesting while heavy-fruiting during on-year season exhausted sugar and carbohydrate reserves in Kinnow plant to simulate low induction of floral buds and hence light fruiting on trees for the coming year (off-season). The fluctuating trend in carbohydrates reserves in citrus cultivars contributes to uneven in fruiting^107^, is not an exclusive phenomenon of citrus^101^ but also observed in avocado^108^ and olive^47^. Fruits load depletes carbohydrate in all plant components^45^ with the leading role of roots carbohydrates of supplying energy for next floral and vegetative buds initiation in citrus^109,110^. In this work, both heavy-fruiting and late harvesting plants were deprived off carbohydrates and energy reserves, especially for Kinnow orchards of TTS and Vehari, in order to impute a more biennial fruiting pattern. In Sargodha, both timely harvesting and reduction of fruit loads by spot picking used in the export of Kinnow fruit have resulted in even fruiting for the next season by maintaining a balance of carbohydrates for both seasonal fruits. Similarly, in Satsuma mandarin (*Citrus unshiu* Marc.), Nishikawa et al.^49^ found more sugar phosphate in light bearing vegetative stems and an additional accumulation of amino acids in heavy-fruiting trees to infer that fruiting habits had a direct impact on coenzymes, sugars and amino acids and had an indirect propensity on flowering and fruit-setting to justify this work. Heavy-fruiting and late harvesting plants have shown more alternative fruiting habits, as observed in this work, equally justified nutritional theory on the accessibility of carbohydrate for next season fruiting^45,51–53,104^ as well as on-tree fruit load inhibitory effect on coming season flowering^50,52,94,102^. The present work is in line with the findings of Monselise and Goldschmidt^28^ that alternate bearings are an innate properties of both early (Satsusma & Michal) and medium-to-late (Kinnow, Murcott, Wilking & Dancy) cultivars with an increasing trend in irregular fruiting in the case of late harvesting.

### Fruit drop trend of Kinnow orchards

Perusal of data regarding fruit drop during on- & off-years of three experimental sites showed significant differences as presented in Table 7. In both fruiting years and fruiting habits, the physiological fruit drops were seen higher in all three districts and the maximum was recorded in Vehari (60%) on heavy-fruiting plants during on-year season and minimum in Sargodha (47%) on light-fruiting plants during off-year period. However, pathological fruit drops were more recoded on heavy-fruiting plants at Sargodha (37%) during on-year and less on light-fruiting plants in Vehari (25%) during off-year. Total fruit drops were seen more on heavy-fruiting plants during the on-year and less on light-fruiting plants in off-year in all three districts. Total fruit drops were recorded as maximum at Sargodha (92%) in heavy-fruiting plants during on-year and minimum on light-fruiting plants in Vehari (76%) during off-year.

**Table 7:** Fruit drop trend at experimental sites during on- & off-years.

Fruit drop	Fruiting habit plants	Fruit drop trend in different fruiting habit plants					
		Sargodha		T.T Singh		Vehari	
		On-year	Off-year	On-year	Off-year	On-year	Off-year
Physiological fruit drop (%)	Heavy fruiting	55 ± 4.23ef	54 ± 3.25f	57 ± 2.88e	57 ± 3.24e	60 ± 3.21e	55 ± 3.11f
	Medium fruiting	52 ± 3.45f	51 ± 2.83f	55 ± 2.71ef	53 ± 3.25f	57 ± 2.14e	54 ± 2.18f
	Light fruiting	48 ± 3.25g	47 ± 4.18g	53 ± 3.21f	49 ± 2.45g	54 ± 3.14f.	51 ± 1.92 fg
Pathological fruit drop (%)	Heavy fruiting	37 ± 3.2h	35 ± 3.11h	34 ± 2.54h	33 ± 3.47d	29 ± 3.45e	32 ± 2.49i
	Medium fruiting	35 ± 3.21h	32 ± 1.73i	33 ± 2.45hi	30 ± 3.13i	28 ± 1.76ij	30 ± 2.74i
	Light fruiting	33 ± 1.92hi	28 ± 4.02ij	30 ± 2.55i	28 ± 2.53ij	26 ± 1.26j	25 ± 3.01j
Total fruit drop (%)	Heavy fruiting	92 ± 3.15a	89 ± 3.24a	91 ± 3.14a	90 ± 3.04a	89 ± 3.11a	87 ± 2.47ab
	Medium fruiting	87 ± 2.98ab	83 ± 3.43bc	88 ± 3.05ab	82 ± 3.23bc	85 ± 2.45b	84 ± 3.14b
	Light fruiting	81 ± 4.21c	75 ± 1.93d	83 ± 3.45bc	77 ± 3.13cd	80 ± 3.12c	76 ± 3.25cd

Results are shown in means (± SD) and sharing different letters are significantly differed to each other according to LSD test (P ≤ 0.05).

Notwithstanding other causes, carbohydrates deficiency contributes to fruit drops in citrus^38^, apple^111^ and sweet cherry^112^ while among other favorable conditions; the availability of carbohydrates also increases flowering as well as fruit set with reduced drops in many fruits, including citrus fruits^113^. Fruits thinning, either natural or artificial, can increase the supply of carbohydrates and further reduce the drop of developing fruitlets^114,115^. Spiegel-Roy and Goldschmidt, 1996^116^ referred to as physiological fruit drop in flowering and developing fruitlets during initial fruit stage, while^43^ concluded that carbohydrates, especially soluble sugars, were available during fruit cell division for fruitlets retention on tree. In Kinnow mandarin physiological fruit drop ranges 40 to 63% and pathological fruit drops from 5 to 25%^117^. Physiological fruit drop is dominant during cell division stage^118^ which lasted for 70–75 days in Kinnow^10^ and has been linked to environment, nutrition and plant-water-balance^119,120^ as well as pest pressure^121^. Similarly, the pathological drop in citrus fruit continued throughout the fruit growth and development stages before harvesting^11,118^ with a dominant effect of adverse weather to proliferate diseases and mature fruit pests such as fruit fly infestation^15^. Same trend of more physiological and pathological fruit drop were seen on heavy bearing plants during on-year and less on light bearing trees in off-years, is in line with previous researchers based on the availability of carbohydrates and developing fruitlets competition for photoassimilates. In addition, dry warm conditions during fruit initial growth stages caused more drops, termed as physiological drop, whereas extended spell of warm-humid conditions in the autumn season has proliferated diseases and resulted in increased pathological drop. Citrus plant physiology was affected by a fluctuating pattern in agrometeorological indices, with different extents of fruit drops seen under climatic variable conditions at three experimental sites, as well as altering the fruiting habit of Kinnow mandarin. Along with weather vagaries^61^, carbohydrate deficiency reduced the induction of floral buds in the coming season^122^ to support the hypothesis that climate variables induce fruit drops as well as alternative fruiting patterns in citrus fruits.

### Yield and fruit grade quality of Kinnow plants

Yield and fruit grade quality of Kinnow plants are significantly differed during both on- & off-years and three different agro-climatic conditions and data are shown in Table 8. Maximum numbers of fruits were harvested on heavy-fruiting plants during on-year (1058 no.) and minimum on light-fruiting plants during off-year (209 no.) at TTS. Fresh fruit weight per plant was recorded as maximum on heavy-fruiting plants during on-year (190.44 kg) at TTS and minimum on light-bearing plants during off-year (36.89 kg) at Sargodha. Higher percentage of A-grade was recorded in the medium-fruiting plants at Sargodha (18.78%) and lower in light-fruiting plants in Vehari (8.5%) during off-year season. However, more B-grade fruits were recorded on heavy-fruiting plants at Vehari (57.8%) and less on light-fruiting plants at Sargodha (38.75%) during off-year. Similarly, maximum C-grade percentage was noted on light-fruiting plants during off-year (55.14%) and minimum on heavy-fruiting plants during on-years (31%) in Vehari district. Higher yields were recorded during on-year at TTS (36,783.8 kg ha^−1^) and lower during off-year at Vehari (15,737.9 kg ha^−1^).

**Table 8:** Yield and fruit grades quality trend at experimental sites during on- & off-years.

Yield parameters	Fruiting habit plants	Yield and fruit grades trend in different fruiting habit plants					
		Sargodha		T.T Singh		Vehari	
		On-year	Off-year	On-year	Off-year	On-year	Off-year
No. of fruit per plant	Heavy fruiting	854 ± 32.23b	714 ± 23.25d	1058 ± 32.8a	867 ± 23.25b	760 ± 23.25c	552 ± 23.7f
	Medium fruiting	524 ± 24.45f	451 ± 22.8g	665 ± 24.7e	551 ± 18.25f	457 ± 12.15g	401 ± 15.8h
	Light fruiting	248 ± 13.5i	217 ± 14.18j	253 ± 20.2i	209 ± 14.4j	224 ± 13.1j	210 ± 11.9j
Fruit weight per plant (Kgs)	Heavy fruiting	136.64 ± 12.36m	121.38 ± 9.87n	190.44 ± 12.48 k	164.73 ± 11.15 l	125.4 ± 5.69n	99.36 ± 8.75o
	Medium fruiting	83.84 ± 8.24p	76.67 ± 7.84p	119.7 ± 8.98n	104.69 ± 9.89o	75.41 ± 6.25p	72.18 ± 7.28p
	Light fruiting	39.68 ± 5.21r	36.89 ± 4.28r	45.54 ± 6.47q	39.71 ± 4.84r	36.96 ± 3.45r	37.8 ± 5.47r
A-grade fruit (%)	Heavy fruiting	16.2 ± 2.2t	18.21 ± 2.31t	14.4 ± 1.5uv	12.3 ± 1.75v	11.2 ± 1.45v	10.25 ± 1.45w
	Medium fruiting	17.41 ± 1.21t	18.78 ± 1.93t	13.2 ± 1.65v	12.87 ± 1.43v	10.25 ± 1.54w	10.65 ± 1.7w
	Light fruiting	15.25 ± 1.42u	16.84 ± 1.82t	12.75 ± 1.65v	11.85 ± 1.91v	9.24 ± 1.46w	8.5 ± 1.6x
B-grade fruit (%)	Heavy fruiting	45.5 ± 5.15q	40.36 ± 3.24q	52.25 ± 4.04q	50 ± 3.54q	57.8 ± 3.51q	54.35 ± 3.27q
	Medium fruiting	43.59 ± 4.98q	42.58 ± 3.43q	48.98 ± 3.4q	52.56 ± 4.25q	55.25 ± 3.45q	51.25 ± 4.1q
	Light fruiting	38.75 ± 3.8r	45.36 ± 1.93q	53 ± 3.52q	47 ± 2.23q	49 ± 2.82q	46.54 ± 3.55q
C-grade fruit (%)	Heavy fruiting	38.3 ± 5.45r	41.43 ± 2.44qr	33.35 ± 2.54s	37.7 ± 2.84r	31 ± 4.21 s	35.4 ± 3.27rs
	Medium fruiting	39 ± 3.48r	38.64 ± 2.73r	37.82 ± 3.45r	34.57 ± 3.23rs	34.5 ± 3.35rs	42.81 ± 3.14q
	Light fruiting	46 ± 3.81q	37.8 ± 2.92r	34.25 ± 3.05rs	41.15 ± 5.23qr	41.76 ± 3.12q	55.14 ± 5.25q
Yield (kg ha^−1^)		26,587.54	16,959.54	36,783.8	20,867.26	25,478.6	15,737.9

Results are shown in means (± SD) and sharing different letters are significantly differed to each other according to LSD test (P ≤ 0.05).

Climate variables have a direct impact on fruit growth and development^10^ and consequently decreased yields with a decline in fruit quality attributes^123^. Holland et al.^124^ recorded a decrease in fruit quality in the era of global warming, while^73^ estimated a 1/4th reduction in citrus yields in the US. In this work, Kinnow fruits of inferior quality were harvested in warm regions during on-and off-years from light, medium and heavy bearing plants. In addition, higher temperatures during the fruit cell division stage led to even more physiological drops^10,11^ resulting in alternate bearings^13^ and finally yielding low-quality fruit^15^. Chelong and Sdoodee^125^ found a direct effect of climate variables on fruit yield and quality in their work on Shogan (*Citrus reticulata* Blanco) in Thailand. In present work, warm dry spells at TTS and Vehari have resulted in a more premature stage Kinnow fruit drops while extending the summer season period has exacerbated stem-end rot disease, causing additional drops in maturing fruits. In fact, both light and heavy fruiting seasons, the fruit drop pattern was related to the external climate, which decreases yield and grade quality while showing further variations in three experimental sites. More declines in yield and grading quality have been seen in light fruiting seasons with high intensity in warm regions. However, during heavy fruiting season, hanging fruits were least affected by the vagaries of weather conditions. Citrus fruit A-grade quality is linked to fruit size, shape and apparent view^15,126^ or blemishes free outer peel^14^, which was more harvested in Sargodha due to timely harvesting and early spot-picking for export while observing less fluctuating climate variables. The physiological mechanism of biennial fruiting trends in citrus^129^ is directly linked with external conditions, especially changing vapor pressure deficit^130^ during fruit growth and development phases^10^ to determine fruit-load for succeeding season. In addition, the relationship between the sources (leaves) photoassimilates and the sink (fruits) also has an effect on the yield and quality of the citrus fruit^127,128^ which has justified this work of recording more A-grade quality fruit on medium bearing trees during on-year. More pest pressure, particularly citrus mites and thrips, was seen in warm areas that directly affected the external outlook of the fruit^11^, reducing the exportable volume^15^, which was recorded higher in warm TSS and Vehari districts to justify less A-grade quality Kinnow fruits produced in more climate variable regions. Present work is in line with previous works on citrus^13,129^.

## Conclusion

Climate variables determine the fruiting habit, yield and quality attributes of Kinnow Mandarin. In warm regions, the fluctuating trend in thermal indices has not only influenced plant phenophases, but also fruiting habits, fruit drops at different stages and, consequently, yield and quality characteristics. In TTS and Vehari, the more unpredictable weather behavior resulting in more variations in thermal indices causes an alternating pattern of fruiting by disrupting the source-sink relationship and deteriorating fruit quality, henceforth affecting plant thermal use energy efficiencies. Biennial fruiting pattern is an inherent character linked to citrus is dominantly induced by external environment has become more prevalent in more climate variable regions as seen in this work. This research endeavor may be fruitful in future to decide particular region regarding citrus fruiting habit, quality as well as yield and also pinpoint major management practices in future where more fluctuations in climate variables arise.
